# Mapping competency profiles of schools of public health: implications for public health workforce education and training in Israel

**DOI:** 10.3389/fpubh.2024.1416497

**Published:** 2024-08-26

**Authors:** Yehuda Neumark, Jordan Hannink Attal, Naham Shapiro, Fiona MacLeod, Janas Harrington, Paul Barach, Jascha de Nooijer, Keren Dopelt, Mariusz Duplaga, Lore Leighton, Hagai Levine, Zohar Mor, Robert Otok, Stephanie Paillard-Borg, Ted Tulchinsky, Shira Zelber-Sagi, Maureen Malowany

**Affiliations:** ^1^Braun School of Public Health and Community Medicine, Hebrew University-Hadassah, Jerusalem, Israel; ^2^School of Public Health, University College Cork, Cork, Ireland; ^3^College of Population Health, Thomas Jefferson University, Philadelphia, PA, United States; ^4^Faculty of Health, Medicine and Life Sciences, School of Health Professions Education, Maastricht University, Maastricht, Netherlands; ^5^Department of Public Health, Ashkelon Academic College, Ashkelon, Israel; ^6^Department of Health Policy and Management, Faculty of Health Sciences, School of Public Health, Ben Gurion University of the Negev, Beer Sheva, Israel; ^7^Department of Health Promotion and e-Health, Faculty of Health Sciences, Institute of Public Health, Jagiellonian University Medical College, Kraków, Poland; ^8^The Association of Schools of Public Health in the European Region (ASPHER), Brussels, Belgium; ^9^The Israeli Association of Public Health Physicians, Israeli Medical Association, Ramat Gan, Israel; ^10^Department of Health Sciences, The Swedish Red Cross University College, Huddinge, Sweden; ^11^Faculty of Social Welfare and Health Sciences, School of Public Health, University of Haifa, Haifa, Israel

**Keywords:** curriculum mapping, competencies, public health education, ASPHER, Israel, schools of public health

## Abstract

**Aim:**

Competency frameworks are essential for analyzing capabilities of Schools of Public Health to adequately prepare public health (PH) professionals to address contemporary challenges. This study maps the competency profiles of PH training programs in Israel using a novel curriculum mapping tool.

**Methods:**

This study assessed all five Israeli Health Education Institutions (HEIs) offering MPH or Bachelors in Public Health (BPH) degrees across 57 competencies in six domains to determine the extent to which competencies were addressed in the curriculum. The competencies list was based on the Association of Schools of Public Health in the European Region (ASPHER) List of Core Competences for the Public Health Professional, adapted for Israeli HEIs.

**Results:**

The core curricula in the four MPH programs addressed 45–84% of all competencies. The BPH program addressed 79% of competencies. In MPH programs, the core curricula addressed most or all competencies in the Methods and the Socioeconomic Determinants of Health domains. Competencies in the domains of Environmental Determinants of Health, Health Policy, Economics & Organization, and Health Promotion and Prevention were less comprehensively addressed in most core curricula. Students’ opportunities to broaden their exposure to competencies outside the core curricula were context dependent.

**Discussion:**

The curriculum competencies mapping tool that was developed served to assess both strengths and shortcomings in PH education in Israel. The findings demonstrate a highly variable array of PH curriculum models in Israeli HEIs, as well as overall shortcomings in the Environmental, Health Policy Economics and Organization, and Health Promotion and Prevention domains. This analysis has already led to reassessment of the curriculum, and will continue to guide the next steps to increase the harmonization of PH training curricula and to better meet PH challenges in Israel.

## Introduction

The recent and ongoing COVID-19 pandemic has centered public health as an essential social good and highlighted the complexities of what constitutes best practice in public health education programs (1, 2). Addressing complex matrices of public health challenges requires qualified, knowledgeable professionals trained in advanced analytic methods and educated in interdisciplinary approaches. Schools of public health (SPH) must maintain relevant and innovative curricula including practical and theoretical tools for research and implementation supported by a robust pedagogical framework, and regularly evaluate their curricula to ensure their programs produce workforce-ready graduates.

Education competency frameworks have emerged over the last decades to address the Lancet Commission Report’s critique that the education of healthcare professionals contains “fragmented, outdated, and static curricula that produce ill-equipped graduates” (3). Competency-based curricula create clear definitions for what knowledge and practices graduates should be able to demonstrate within a professional setting, rather than merely displaying adequacy in meeting institutional instructional goals (4). However, clear methodologies for applying competency frameworks to existing public health curricula are scarcely available, save within the context of accreditation (5).

The aim of this study was to assess current Israeli public health curricula using a novel competency-mapping tool based on the ASPHER European List of Competences for the Public Health Professional (6). The outcome of this aim is twofold: first, this article highlights assets and shortcomings of the current Israeli public health curricula identified through curriculum mapping of required and elective courses; Second, this article records the process of using a competency mapping tool on a national scale, including implementing location-specific adaptation. This description may offer insights to curriculum developers internationally concerning the methodology of evaluating curricula using a competency framework.

## Methods

### Study background

#### Study setting and participants

The study was carried out through the multi-national European Union funded Erasmus+ “Sharing European Educational Experience in Public Health for Israel: Harmonization, Employability, Leadership and Outreach” (SEEEPHI) project. The project was initiated to align Israeli public health programs’ curricula design to European competency standards, increase comparability among Higher Education Institutions (HEIs), and align Israeli public health education to meet local labor market needs (7–9). SEEEPHI Work Package 3 (WP3) was tasked with mapping the competency profiles of Master of Public Health (MPH) and Bachelor of Public Health (BPH) programs offered by Israeli HEIs to identify areas of focus and potential gaps in curricula. This work was carried out jointly by research teams at The Hebrew University of Jerusalem (HUJI), Israel and University College Cork (UCC), Ireland.

Four Israeli universities (HUJI, Haifa University, Ben Gurion University of the Negev, Tel Aviv University) offer MPH programs, and one college (Ashkelon Academic College) offers a BPH degree. All five HEIs participated in the study. Each HEI appointed a study focal person, usually a senior faculty member with department leadership experience, who oversaw completion of the questionnaire.

#### Questionnaire development

We developed and validated a questionnaire based on the European list of WHO-ASPHER Competencies Framework for the Public Health Professional (6). The ASPHER core competencies list comprises a detailed inventory of competencies across six generic domains: Methods in Public Health (Methods); Socioeconomic Determinants of Health (SES); Environmental Determinants of Health (Environment); Health Policy, Economics and Organization (Policy and Econ); Health Promotion and Prevention (Promotion); and Ethics. A condensed list was created by collapsing the list of 129 competencies and sub-competencies into 57, while retaining the overarching six-domain structure (Appendix 1).

The mapping tool contains measures of theoretical and applied learning of competencies within curricula. Respondents were asked to indicate the number of core and/or elective courses in which the theoretical aspects of each competency are addressed. To assess applied learning opportunities, respondents were instructed to indicate which, if any, of the following learning opportunities was available to students for each competency: course exercises, course exam, thesis, capstone project or paper, workshop, and fieldwork.

The tool was completed separately for each Master/Bachelor of Public Health program offered by the participating HEI and for each specialization (the sub-focus chosen by students at or after enrollment) within the program.

Additionally, respondents were asked to describe the program structure for each specialization and the number of graduates of each specialization in the past 5 years.

Respondents were also asked to rank the relative coverage of each competency addressed by the program’s curriculum on a Likert-type scale ranging from 1 (very low coverage – mentioned in one or more courses but not necessarily accompanied by an exercise or exam questions) to 4 (very high coverage—addressed in detail in one or more courses and accompanied by an exercise and/or exam questions).

#### Data collection

Following development, piloting and refinement of the questionnaire, the questionnaire was distributed to the focal persons at the participating HEIs. A member of the research team was available to answer questions and assist in completing the questionnaire.

#### Data analysis

Analysis focused primarily on competencies addressed and their relative coverage, stratified by HEI and by specialization. Each MPH specialization was analyzed individually and subsequently compared to other MPH specializations within the same HEI and to equivalent specializations offered by other HEIs.

Program structures were analyzed to determine the respective core and elective curricula of each specialization and track. This analysis accounted for research and non-research tracks in MPH programs. Research tracks typically require students to complete a thesis while non-research tracks require students to complete a final paper or capstone project, or pass a comprehensive exam.

Each track and specialization were analyzed for the following components: *core courses* (mandatory courses in which all students studying within the program are required to enroll regardless of their track or specialization), *specialization core courses* (mandatory courses for all students studying in the specialization), *specialization elective courses* (or “restricted electives,” are courses chosen by students from a list of preferred elective courses for a specialization), and *open elective courses* (elective courses that all students enrolled in the program can opt to take, regardless of specialization or track).

Whether or not a competency was addressed was coded dichotomously—addressed by one or more courses or not addressed in any course. For each of the six domains, the total number and percent of competencies addressed was calculated separately for core courses, elective courses, and all courses. A competency that was addressed by both a core and elective course was only counted once in the all-courses calculation. For each track and specialization, a summative total of competencies addressed was calculated and expressed as a percent of the 57 competencies in the abridged list.

Relative coverage for each competency was analyzed by calculating the proportion of competencies addressed within a course that received a high (3 or 4) or low (1 or 2) relative score for each program. For MPH programs, relative coverage was calculated for both core and overall course curricula. A competency was considered to have an applied learning opportunity if one or more applied learning opportunities were endorsed.

Competencies addressed were further analyzed across all HEIs offering an MPH program, adjusting for the number of graduates in each program. These data represent the collective educational output of the HEIs, controlling for the wide-ranging graduation figures between the various specializations and tracks. The overall median number of graduates was imputed for the one university that did not provide information about their number of graduates.

All results are de-identified, because in Israel as elsewhere, there is an inherent inter-HEI competition for prestige and training budgets which are directly tied to student enrollment.

## Results

Between 2017 and 2022, four of the five participating HEIs reported producing 746 MPH graduates and 80 undergraduates (one HEI did not disclose graduation figures). Among the three MPH programs that reported the number of graduates per specialization, 29% specialized in health systems, 25% specialized in health promotion, 16% studied in a non-specialization track, and less than 10% specialized in infectious diseases prevention, environmental health, epidemiology, or in mother and child health.

### Public health program structures and core curricula

#### Undergraduate program

The undergraduate program is a three-year full-time program leading to a Bachelor of Public Health degree. All courses in the curricula are mandatory and total 120 credits.

#### Master of public health programs

While there are similarities between MPH program structures, their differences play a key role in each program’s addressing of competencies across all domains.

The four MPH programs are structured to facilitate student employment during their studies. Students commit 1 or 2 days of study per week over 2 or 3 years.

The MPH core curriculum in three programs comprises 10–11 courses (20.5–24 credits) and 4 courses (8–10 credits) in the other program (Table 1). All four programs offer a research (thesis) track and a non-research track. To qualify for the research track, students must meet university and program-specific requirements, typically based on academic performance in the first academic year. For research track students, additional core courses, primarily in advanced statistics and a research seminar, are required. For three of the four programs, these additional core courses pose a restriction on elective course enrollment. One program demands fewer core courses, enabling research track students to enroll in more elective courses. Institutions differ in their approach for requirements for non-research track students. As seen in Table 1, two programs required additional core courses for non-research track students, one required a mandatory guidance course on final projects, and one program had no additional core requirements for non-research track students.

**Table 1:** Core curricula of MPH by HEI*.

Competency domain	HEI 1		HEI 2		HEI 3		HEI 4	
	Course name	Credits**	Course name	Credits	Course name	Credits	Course name	Credits
Methods in public health	Epidemiology 1	3	Epidemiology and Research Methods 1	2	Intro to Epidemiology	2	Epidemiology and Research Methods	6
	Epidemiology 2	3	Epidemiology and Research Methods 2	2	Survey & Research Methods in Epidemiology	2		
			Interpretation of Epidemiological Data	3	Epidemiological Research Planning and Writing Research Proposals	1		
	Biostatistics 1	3	Statistical Methods for Public Health	4	Biostatistics A and Computer Lab	4	Biostatistics 1	2
	Biostatistics 2	3			Biostatistics 2	3		
	Intro to SPSS	2						
Social determinants of health	Intro to Sociology of Health	2	Sociology of Health and Illness	2	Psychosocial Aspects of Health and Illness	2		
Environmental determinants of health	Intro to Environmental Health – OR – Intro to Health Promotion	3	Environmental and Occupational Health	2				
Health policy, economics, and organization	Health Systems in Israel – OR – Intro to Health Economics	3	Organization & Management: Public Health Services	2	Intro to Health Systems Administration	2		
			Health Economics	2	Introduction to Health Economics	2		
Health promotion	Intro to Health Promotion – OR – Intro to Environmental Health	3	Health Promotion	3				
Ethics					Ethics of Medical Research	0.5		
Miscellaneous	Intro to Public Health	2	Intro to Public Health	0	Public Health: From Theory to Practice^***^	2	Intro to Public Health^***^	2
	Knowledge of the law and regulations against sexual harassment (tutorial)	0			Library Resources for the Biological and Medical Sciences	0	Scientific Writing	0
Total core courses	10 courses	24	10 courses	22	11 courses	20.5	4 courses	10

* MPH core courses consist of the mandatory courses in which all students studying within an HEI are required to enroll regardless of their track or specialization.

** We refer to all program structures as using “credits.” While some HEIs use “credits” and others use “semester hours,” the meaning is consistent across HEIs: 1 credit is equal to 1 h of class time per week over the course of a semester.

*** HEI 3 and HEI 4 report that “Introduction to Public Health” and “Public Health: From Theory to Practice” courses address subject matter in the “unaddressed” domain.

Specializations are offered in all four MPH programs, although specialization curricula are approached differently across institutions. These differences are notable because they significantly affect students’ exposure to competencies. Most programs include specialization-specific core courses, which curtails the number of open electives students can take. Specialization-specific requirements regarding elective options compound research/non-research track requirements, further restricting time and credit allowances for open electives. Specializations in health systems, environmental health, epidemiology and biostatistics, and health promotion were offered in most programs (Table 2).

**Table 2:** Specialization tracks and applied learning opportunities offered in Israeli MPH programs.

		HEI 1*	HEI 2	HEI 3	HEI 4*
Health systems/policy	Specialization	✓	✓	✓	✓
	Applied learning opportunity		Workshops		Workshops + Fieldwork
Health promotion	Specialization	✓	✓	✓	✓
	Applied learning opportunity		Workshops	Workshops + Fieldwork	Workshops + Fieldwork
Environmental health	Specialization	✓	✓	✓	✓
	Applied learning opportunity		Workshops		Workshops
Epidemiology and biostatistics	Specialization		✓		✓
	Applied learning opportunity				Workshops + Fieldwork

* HEI1 and HEI4 offer additional specializations (e.g., infectious disease prevention and mother and child health) that are not offered by the other HEIs.

### Competencies addressed and their relative coverage

#### Undergraduate program

The undergraduate program addresses 45 competencies (79%), notably including all competencies in the Methods, SES, and Ethics domains, and all but one of the competencies in the Promotion domain. There is less focus on the Policy & Econ and Environment domains, with 60 and 36% of these domains addressed, respectively. Of the 45 competencies addressed, 35 (78%) are covered within one or more courses as well as class exercises and/or exams, scoring a high relative coverage score.

For MPH programs, the competencies addressed and their relative coverage are reported in terms of core curricula, elective courses, and overall curricula, across HEI.

#### MPH programs-core curricula

**Figure 1:**
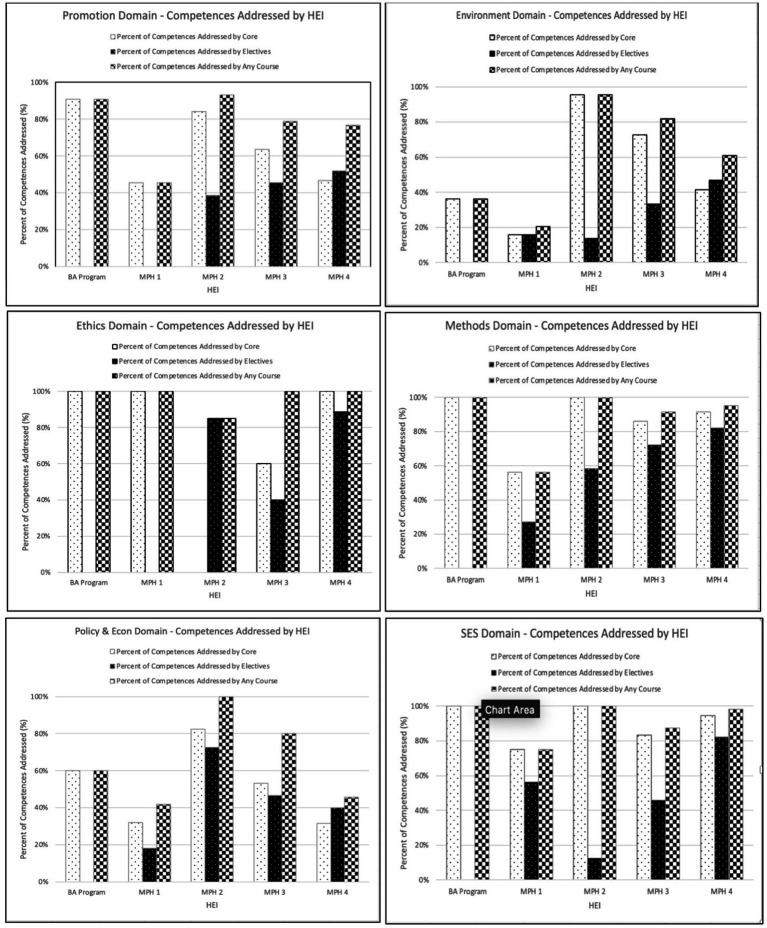
Public health domain competencies addressed by core, elective, and all courses across all specializations of each HEI, Israel.

The percentage of competencies addressed by core courses varied substantially across MPH specializations, ranging from 45 and 84%. The majority of Methods and SES domains’ competencies were addressed by all programs, earning an overall weighted coverage of 79 and 85%, respectively. The other domains were addressed to a lesser extent in all MPH programs, with greater variance across programs, particularly in the Environment domain, in which the percentage of competencies addressed ranged from 16 to 95% and the overall weighted coverage was 44%. Less variation was observed in the Policy and Econ (weighted overall: 46%; range: 32–83%) and Promotion domains (weighted overall: 57%; range: 45–84%). With the exception of one program, all MPH programs address Ethics domain competencies in their core curricula, cumulating in 74% overall weighted coverage of the Ethics competencies (range: 0–100%). A visual summary of domains addressed by core curricula across HEI is presented in Figure 1.

The relative coverage of competencies addressed likewise varies across programs. Two of the MPH programs addressed all of the competencies in detail, scoring a relative coverage score of 69%, and two programs in considerably less detail, scoring a lower relative coverage score (53%).

#### MPH programs-elective courses

Programs with fewer competencies addressed by the core curriculum reported greater proportions of competencies addressed in their elective offerings. One program, which demands fewer core courses and addresses fewer competencies in their core curriculum, addressed 63% of competencies in their elective courses, and over 80% of those in the Methods, SES, and Ethics domains. All other MPH programs addressed less than half of the competencies in elective courses. The distribution of domains addressed differed by program.

#### MPH programs-overall curricula

Overall, most HEIs offered comprehensive curricula, with two programs addressing >85% of competencies across their core and elective course offerings. The level of coverage of the addressed competencies was similarly high in both programs (92–100%). Considerable variability in the percentage of competencies addressed was reported by one program, with 95–100% of competencies addressed in the domains of Methods, SES and Ethics, compared with 61, 46, and 77% in the Environment, Policy and Econ, and Promotion domains, respectively. This program similarly reported a high percentage (77%) of competencies addressed with high relative coverage across the curriculum. Another program addressed less than half of competencies (47%) across all courses. This program particularly focused on SES (75%) and Ethics domains (100%) and to a lesser extent Methods (56%). In this program, more than half of the competencies addressed merited a low relative coverage score. Figure 1 provides a visual summary of domains addressed by core, elective, and any course across HEIs.

**Figure 2:**
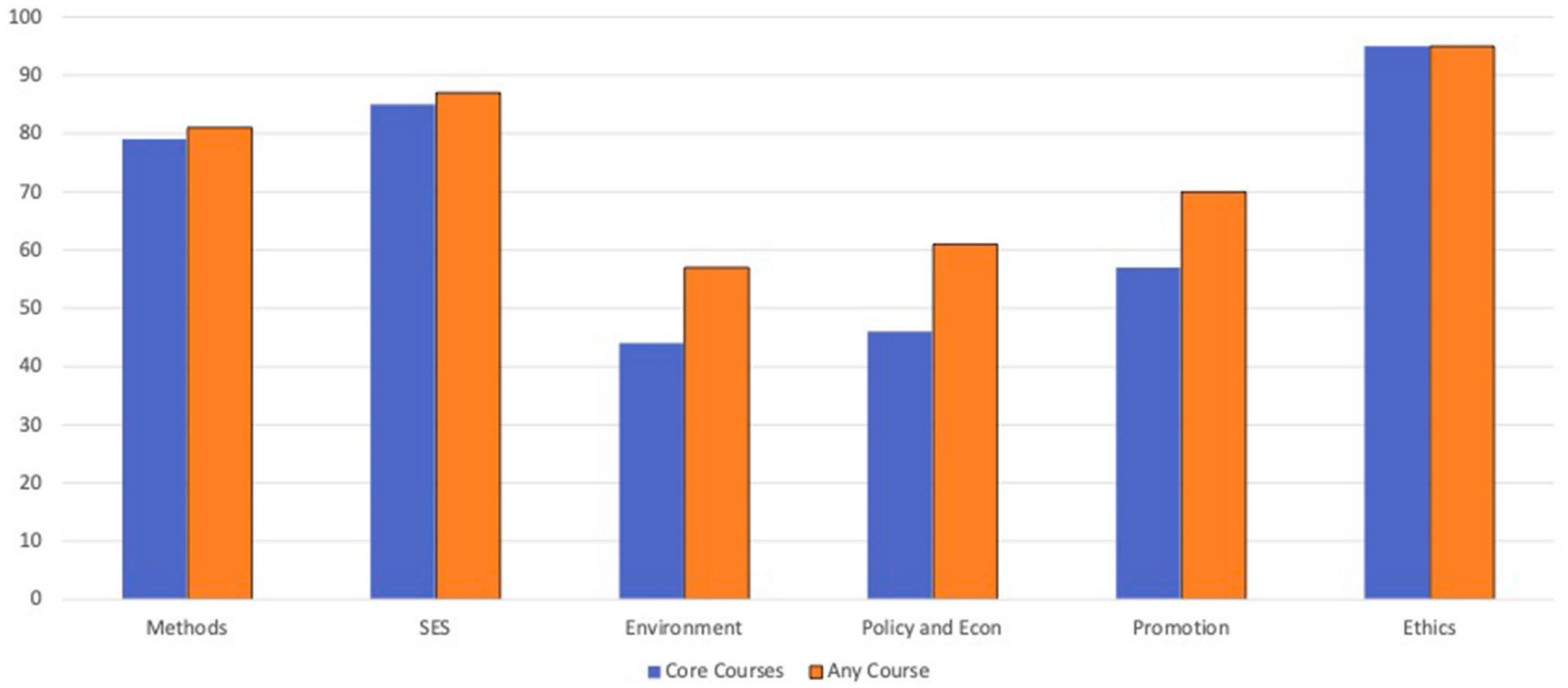
Percentage of competencies addressed by Israeli MPH programs, adjusted by graduates, by domain.

When looking holistically at the core and elective offerings of HEIs with MPH programs and adjusting for the number of graduates, the Ethics (95%), SES (87%), and Methods (81%) domains were well covered, whereas the Promotion (70%), Policy and Econ (61%), and Environment (57%) domains were less well covered. Figure 2 presents the adjusted percentages of competencies addressed by MPH programs in the core and overall curricula.

### Applied learning opportunities

#### Undergraduate program in public health

In the undergraduate program, applied learning took several forms. First, students are expected to apply skills learned during the final practicum, and may include conducting a research project, designing a health promotion intervention, or carrying out a survey. Second, students also partook in varying degrees of fieldwork in the Methods, SES, Policy, and Econ, and Ethics domains. Third, some of the mandatory courses in the final year were integrative, in which students are expected to apply learned principles to a public health issue during in-class exercises.

#### MPH programs

Applied learning opportunities varied between MPH programs and across specializations. A central tenet was that all students were required to apply their knowledge and skills in writing a thesis or final project. The HEI contact persons reported that all competencies not addressed within courses (specifically those related to program implementation and analysis, research protocol development, and research implementation) were addressed and covered in-depth through extensive mentoring during thesis/project preparation. In other respects, variation was noted between the HEIs. For example, one HEI offered numerous applied learning opportunities in the form of workshops and fieldwork, whereas others did not. This variation was particularly stark between specializations. Table 2 describes applied learning opportunities in the different specializations.

## Discussion

“*A curriculum is a delicate plant; it will thrive only with constant attention, proper nourishment and periodic trimming and shaping*” (10).

Our findings highlight the importance and operationalization of a competency framework for public health curriculum assessment by applying a validated tool to evaluate Israeli SPH curricula based on the ASPHER List of Core Competences for the Public Health Professionals (6). As highlighted by the above quote from Kahn and Tollman et al. (10), public health competency frameworks provide benchmarks for best practices in public health education and a potential basis for sharing of core content across and between programs. While there are several descriptions of competency frameworks in public health education design (11, 12), few publications detail the methods and results of applying such frameworks to existing curricula (5, 13–15). The present study describes the development of a competency mapping tool and its application to differentiate between competency domains addressed and their level of coverage for core and elective curricula, for research and non-research tracks, across specializations and across HEIs.

It is of methodological import that differences in program structure compounded with student choice of specialization and track, make direct comparisons between HEIs difficult. Two of the four HEIs offering MPH programs addressed a high proportion of competencies in their core curricula with high relative coverage levels. The competencies to which all students are exposed in the core curricula, regardless of specialization or research track, are the central measure of common competencies of the new generation of public health graduates. Using the adjusted overall coverage score enabled the estimation of academic preparedness of all MPH graduates on the national level from a curriculum planning perspective.

The key findings of this study indicate that, in its totality, Israeli public health education thoroughly addressed competencies in the Methods and SES domains, adequately addressed competencies in the Ethics domain (save for one HEI) while competencies in the Promotion, Policy and Econ, and Environment domains were inadequately addressed. As stated above, one of the aims of the SEEEPHI project in its entirety is to align Israeli public health education to meet local labor market needs. A parallel work package in the SEEEPHI framework addressed workforce competencies required by employers. Necessary competencies differed by organization type, wherein the largest deficiencies were reported by research institutes, including in Science and Practice, Promoting Health, Law, Policy, and Ethics, and One Health and Health Security (16). These workplace competencies overlap with academic competencies in the Promotion, Policy and Econ, and Environment domains, and serve as an indirect indication that inadequately addressed competencies in the aforementioned domains are translating to the workforce. Work has been initiated to more fully explore and address the education-workforce gaps. Furthermore, looking upstream, our results are informing the development of an online public health professional “marketplace” platform that is currently being developed and piloted by another SEEEPHI Work Package. The goal of this marketplace is to facilitate direct communications between employers, HEIs, potential students, and graduates. It is anticipated that the totality of the SEEEPHI outputs will ensure that the Israeli public health workforce is suitably educated prior to entering practice.

Some inter-program variability in competencies addressed is expected given the diversity of public health-related occupations. The discrepancies in competencies addressed in core curricula of the programs reviewed may reflect pedagogical differences in the relative merits of “breadth” vs. “depth” as well as the relative importance of student autonomy within the framework of a structured curriculum. These discrepancies may, however, merely reflect the evolution of curricula over time as they adapted to changes in prevailing public health topics, expertise of lecturers, or administrative policies. Further work is needed to explore in greater depth the differences in curricula foci and their underpinnings among the participating HEIs.

Likewise, it is important to note that the ability of students to enroll in elective courses and benefit from-- or forfeit-- exposure to additional competencies largely depends on the program’s structure. For example, two programs that addressed a relatively high percent of competencies in their core curricula, significantly limited elective options due to their research track and specialization requirements, and thereby impinging on students’ ability to diversify their range of competencies.

Most recent graduates specialized in health systems or health promotion. The division of students by specialization may reflect their existing career tracks, or their envisioned “next steps” in training or career advancement. Given differences in specialization curricula within and between MPH programs, students specializing in health systems or health promotion, though benefiting from increased exposure to the competencies addressed in these domains, may be missing competencies in other domains even while these competencies may receive high relative coverage in their HEI’s elective curricula.

In light of the growing threat that the climate crisis poses to public health, especially in Israel and other countries in the Middle East, the low coverage of competencies in the Environment domain is distinctly concerning. The inadequate coverage of competencies included in the Environment domain will be analyzed in a future paper.

Over the last two decades, undergraduate education in public health has gained traction in the United States and Europe (17–19). Israel followed this trend in 2014, opening its first BPH program (20). The program is composed of exclusively core courses, and addresses a substantial proportion of competencies with high relative coverage. Despite the strength of the undergraduate program, data from other countries suggest that there are limited workforce opportunities for BPH graduates, particularly in government positions (19). This concern is likewise relevant for Israeli BPH graduates, especially for BPH holders who do not hold diplomas in a paramedical profession (19).

### Strengths and limitations

This survey, to the best of our knowledge, is the first systematic competency-mapping of public health education in Israeli HEIs. As all relevant HEIs participated, the results reflect the totality of MPH and BPH students in the country. Yet, as only BPH and MPH programs were assessed, to the exclusion of other public health relevant programs (e.g., Masters in Health Administration), the results presented provide an incomplete picture of public health education in Israel.

The findings have contributed to strengthening public health education by serving as a measure of academic offerings relevant to professional practice in Israel. In turn, HEIs will be empowered to adjust educational programs and create greater alignment between educational offerings and workforce needs in the coming years. The process of curriculum mapping and the presentation of the results to all the SPHs served as catalysts for addressing shortcomings, which represent a major contribution of the curriculum mapping process to academic planning. In addition to adjustments and alterations to individual HEI curricula, the overall results of the curriculum mapping were well-received by the Council of Higher Education of Israel, which contributed to support for amplifying and strengthening public health curriculum across institutions.

Our study reflects the context and distinct constraints of Israeli PH education, which differ from other countries, although the methodological rigor and robust research design should be applicable and generalizable to other countries. The application of the mapping tool can serve, therefore, as a template for SPHs in Europe and beyond.

The findings, however, must be interpreted in light of several limitations inherent in the mapping tool developed and its application in the local Israeli context. First, while the survey mapped the competencies that appear in the program curricula, it could not assess the level of complexity or depth to which each competency is addressed. As such, caution is advised in interpreting these results to compare the quality of education across HEIs. Nor, obviously, does the tool assess the extent to which competencies were “learned” and internalized by the students. A second-step of evaluating HEIs should address the introduction, development, and end-point knowledge of competencies among students in order to better assess the quality of instruction and graduates’ preparedness for the current and future public health workforce. Second, there was a fair degree of variability across HEIs in how the survey instrument was completed. Participating HEIs were instructed to appoint, for each competency domain, a focal person familiar with the material taught in the relevant core and elective courses and workshops. Some HEIs appointed a single contact person to complete the survey for all domains, others appointed multiple focal persons or requested each lecturer to complete the survey for her/his course(s) and subsequently combined the information. Due to differences in how HEIs completed the survey, caution is advised in the comparison between HEI programs, tracks, or specialties. Third, as with a study of ASPHER member schools (21), the tool used in this study does not readily allow for direct comparisons across programs due to the structural variations between HEIs. However, this study provides a template for SPH to identify curriculum differences with the aim toward harmonizing public health education.

## Conclusion

The present study considerably advances the potential benefits and challenges of operationalizing a competency framework for public health curriculum assessment. The mapping tool provides practical means to apply competency frameworks to existing public health curricula to describe the potential “toolbox” that graduates take with them into the workforce. The ASPHER List of Core Competences for the Public Health Professional served as a useful framework, yet adaptation of the tool to a local context is necessary in order to produce meaningful and actionable results HEI leaders. Turning globally, this study’s findings support the need for operationalizing regional or international competency frameworks with the flexibility necessary to respect country-specific contexts. Further work is necessary to examine how educational and workforce competency frameworks can be better harmonized, to develop more effective strategies in narrowing the gaps between public health curriculum delivery, competency acquisition by students, and real-work practice.

## Data Availability

The raw data supporting the conclusions of this article will be made available by the authors, without undue reservation.
