# Supplementary material for: Mapping competency profiles of schools of public health: implications for public health workforce education and training in Israel
Source: Front Public Health. 2024 Aug 26;12:1416497. doi: 10.3389/fpubh.2024.1416497 (PMC11381267; doi:10.3389/fpubh.2024.1416497)
Supplement: Supplementary file 1 [file Table_1.docx]

# **Appendix 1:** Abridged ASPHER's European List of Core Competences for the Public Health Professional

| **Domain** | **Competences** |
| --- | --- |
| Methods | Definitions of Health & Public Health |
|  | Disease Classification Systems |
|  | Basic Epidemiologic/Demographic Concepts |
|  | Epidemiologic Study Designs: Observational |
|  | Epidemiologic Study Designs: Experimental |
|  | Qualitative Research Methods |
|  | Questionnaire Design & Scaling |
|  | Statistical Concepts |
|  | IT & Data Handling |
|  | Scientific Writing & Reading |
|  | Develop a PH Research Protocol |
|  | Conduct a PH Research Project |
| SES | Basic Population Health Definitions & Concepts |
|  | Population Health Indicators |
|  | Socioeconomic Determinants - Concepts |
|  | Socioeconomic Determinants - Indicators |
|  | Health Behavior - Indicators |
|  | Models and Theories of Social Determinants of Health |
|  | Develop a Population Health Research Protocol |
|  | Conduct a Population Health Research Project |
| ENVIRONMENT | Basic Environ. Health Definitions & Concepts |
|  | Environmental Health Exposures – Risk Factors |
|  | Environmental Measurement / Surveillance |
|  | Governance and Stakeholders |
|  | Food Security / Food Safety |
|  | Emergency Planning/Management |
|  | Preventing and Controlling Hazards |
|  | Conduct Risk and Health Impact Assessments |
|  | Develop Environmental Health Strategies |
|  | Develop an Environ. Health Research Protocol |
|  | Conduct an Environ. Health Research Project |
| POLICY & ECON | Basic Economic Theory and Concepts |
|  | Basic Organizational Theory; Leadership and Management Concepts |
|  | Evaluation Strategies and Designs – Impact Evaluations |
|  | Evaluation Strategies and Designs – Health Economic Evaluations |
|  | Evaluation Strategies and Designs – Organizational/Program Evaluations |
|  | Evaluation Strategies and Designs – Policy Evaluations |
|  | Disaster Management |
|  | Program Implementation and Analysis Strategies |
|  | Develop a PH Policy/Strategy/Intervention Project |
|  | Implement a PH Policy/Strategy/Intervention Project |
| PROMOTION | Definitions of Health Promotion, Protection, & Disease Prevention |
|  | Basic Health Promotion Concepts |
|  | Major Theories and Models |
|  | Principles of Health Communication |
|  | Health Promotion Program Design & Methods |
|  | Emergency Planning and Management |
|  | Health Promotion Program Research Design |
|  | Program Implementation and Analysis Strategies |
|  | Develop a Health Promotion Policy Proposal |
|  | Develop a Health Promotion Project |
|  | Implement a Health Promotion Project |
| ETHICS | Basic Ethical Theories and Concepts |
|  | Ethical Aspects of Public Health Strategies, Interventions, and Policies |
|  | Ethical Aspects of Public Health Research |
|  | Data Protection and Storage |
|  | Ethics Committee Systems |
